# The Association Between Informal Caregiving and Exit From Employment Among Older Workers: Prospective Findings From the UK Household Longitudinal Study

**DOI:** 10.1093/geronb/gbw156

**Published:** 2016-12-06

**Authors:** Ewan Carr, Emily T Murray, Paola Zaninotto, Dorina Cadar, Jenny Head, Stephen Stansfeld, Mai Stafford

**Affiliations:** 1 Department of Epidemiology and Public Health, University College London, UK; 2 Wolfson Institute of Preventative Medicine, Queen Mary University of London, UK

**Keywords:** Caregiving, Extended working, Understanding Society, Random effects logistic regression

## Abstract

**Objective:**

This study investigated associations between informal caregiving and exit from paid employment among older workers in the United Kingdom.

**Method:**

Information on caregiving and work status for 8,473 older workers (aged 50–75 years) was drawn from five waves of Understanding Society (2009–2014). We used discrete-time survival models to estimate the associations of caring intensity and type on the probability of exiting paid work (from >0 to 0 hours/week) in the following year. Models were stratified by sex and working hours, and adjusted for age, self-rated health, long-standing illness, occupation, and partner’s employment status.

**Results:**

No association was found between caregiving intensity and exit from paid work. Full-time employees who provided care within the household (women and men) or cared for a partner/spouse (women only) more likely to stop working, compared to those not providing care. Women who entered a caregiving role (more than 10 hours/week) were between 2.64 (95% confidence interval [CI]: 1.46, 4.79) and 4.46 (95% CI: 2.53, 7.88) times more likely to exit work (for part-time and full-time workers, respectively), compared to women providing no care.

**Discussion:**

This study highlights the onset of caregiving as a key period for older workers. Ensuring that caregiving responsibilities are adequately recognized and supported may help extend working life.

Life expectancy at birth has risen remarkably in the last 30 years, mainly due to improvements in mortality rates at older ages (Dunsmith & Large, 2016). As a result, in many countries, the proportion of older people is sharply increasing. In the United Kingdom, most adult social care is provided informally as unpaid care from family or friends, with over 5.8 million people engaged in a caregiving role in 2011 (Robards, Vlachantoni, Evandrou, & Falkingham, 2015). Demand for informal care is forecast to outstrip supply by 2017 (Pickard, 2015) and is growing at a time when formal provision is declining. Net expenditure (Net current expenditure is total expenditure excluding capital charges and less all income.) on adult social care in the United Kingdom decreased by 11.4% between 2010 and 2015, from £14.6 to £12.9 billion (Burchardt, Obolenskaya, & Vizard, 2015).

Older workers are increasingly expected to provide care for partners or relatives, at a time when governments are seeking to extend their paid employment (up to and beyond statutory pension age). Caregiving is predictive of poor mental and physical health outcomes (Pinquart & Sörensen, 2003) which may subsequently lead to early labor market exit (van Rijn, Robroek, Brouwer, & Burdorf, 2014). Increasing participation in informal caregiving has elsewhere been shown to reduce the labor supply of older workers (Harper, 2004). It is therefore important to understand the impact of caregiving on paid work beyond age 50.

## Conceptual Frameworks Relating Caregiving to Paid Work

Many past studies have adopted a microeconomic perspective, framing the relationships between caregiving and paid work as a question of individual time allocation. This approach emphasizes the cost of foregone earnings due to caregiving responsibilities (Carmichael & Charles, 2003). Individuals seek to maximize “utility,” which can be derived from pecuniary (e.g., earnings from paid employment) and nonpecuniary roles (e.g., the “emotional returns” of caregiving; Spiess & Schneider, 2003). Since time is finite, each additional hour of caregiving reduces the time available for other activities, both paid (i.e., employment) and unpaid (e.g., leisure or volunteering). Facing demand for care from a partner or relative individuals may choose to reduce their working hours or exit paid employment altogether.

The decision to reduce working hours in response to caregiving demands will depend on the marginal utility of paid work. Workers receiving low wages are more likely to replace hours of employment with caregiving, compared to workers receiving a higher wage, for whom the lost earnings would be greater (Carmichael, Charles, & Hulme, 2010). Similarly, caregivers providing a small proportion of household income may be more likely to reduce work hours, compared to household members who are the main breadwinner (Mentzakis, McNamee, & Ryan, 2008). Work may also provide nonpecuniary forms of utility, such as self-esteem, support from coworkers, or enjoyment (Carmichael & Charles, 2003), and may offer a respite from caregiving responsibilities (Mooney, Statham, & Simon, 2002). Workers who derive nonpecuniary benefits may prefer to protect their working hours, at the expense of other roles such as leisure. Conversely, workers who are dissatisfied with their job “may see caregiving demands as a good reason to leave an unrewarding job” (Pavalko & Artis, 1997, p. S171).

Other studies have emphasized conflicts between work and family roles (Gordon, Pruchno, Wilson-Genderson, Murphy, & Rose, 2012). Caregiving tasks that can be carried out alongside work obligations are likely to be less strongly associated with labor market withdrawal, and vice versa. This will depend on the type of care provided as well as characteristics of the caregiver’s job. Caregiving roles involving “nonshiftable” tasks (Hassink & van den Berg, 2011), that must be carried out at particular times in the day, may be harder to combine with paid employment than “shiftable” care tasks, which can be done at any time. Similarly, jobs with rigid or unpredictable working schedules will be harder to combine with intensive caregiving roles, compared to more flexible employment arrangements.

## Past Evidence on Caregiving and Extended Working

The growing literature on caregiving and extended working has produced mixed results, with some studies finding a negative association between caregiving and paid work, but others finding no effect. Harper (2004) found women (aged 53–63 years) providing personal care for parents tended to work fewer hours (751 per year) compared to those not providing personal care. van Houtven et al. (2013) found caregivers (aged 50–70 years) to be less likely to be working (men) and more likely to retire (women), compared to noncaregivers. Brown et al. (2014) found women (aged 55 years) providing more than 10 weekly hours of care were less likely to be in full-time employment. Other studies have been less supportive. Ciani (2012) found a weak negative association between caregiving and the probability of being employed (ages 40–64 years), whereas Mooney et al. (2002) found no association between caregiving and employment status beyond age 50. Leigh (2010) found the impact of caregiving on labor market participation to be “small or nonexistent” (p. 9).

Some studies have found a positive association between caregiving and paid work. King and Pickard (2013) found that women older than 50 years who started providing low-intensity care (<10 hours/week) were more likely to remain in employment (compared to noncaregivers), whereas Kubicek et al. (2010) found workers (aged 53–67 years) providing spousal care were less likely to opt for early retirement. Others have demonstrated the reverse association, that is, a negative relationship between paid employment and subsequent caregiving (Carmichael et al., 2010; Kotsadam, 2011). Several studies have suggested a bidirectional care–work relationship (Pavalko & Artis, 1997).

Our first research question, therefore, is whether hours of caregiving per week are associated with subsequent withdrawal from paid employment. Past studies suggest caregiving of 10 or more hours/week is predictive of work exit (Carmichael & Charles, 2003; Ruhm, 1996), whereas caregivers providing fewer hours are no less likely to be working than noncaregivers. Ettner (1995) describes a “threshold effect,” such that when “caregiving responsibilities are minimal, women may be able to juggle employment and caregiving by reducing work hours” (p. 73).

Our second research question asks whether this relationship depends on the type of care provided. Several studies have shown the negative effects of caregiving on employment to be limited to coresident caring roles (Corti, Laurie, & Dex, 1994; Heitmueller & Michaud, 2006). Such roles may reflect higher caregiving demands (Nguyen & Connelly, 2014) or involve tasks that are more time and energy intensive (e.g., personal care). Coresident caregivers may also have less choice about whether to provide care, compared to extraresident caregivers. The caregiving relationship is another potential moderating factor, with previous research showing partner/spousal care to be more strongly associated with work exit, compared with care provided for friends or other family members (Dentinger & Clarkberg, 2002). Our third research question tests whether changes in caregiving responsibilities are predictive of work exit. Past research suggests older workers entering a caregiving role are at increased risk of reducing work hours (Pavalko & Artis, 1997; Spiess & Schneider, 2003) or stopping work (King & Pickard, 2013), compared with those continuing an existing role.

Our fourth research question tests whether these relationships differ between women and men. Many studies have emphasized the gendered nature of caregiving decisions. Throughout the life course, women are more likely than men to provide informal care (Arber & Ginn, 1995), except at the oldest ages (more than 65 years), where men are more likely to provide care (Vlachantoni, Evandrou, Falkingham, & Robards, 2013). Compared to men, women are more likely to be the primary caregiver (Allen, 1994), to care for a spouse or parent (Neal, Ingersoll-Dayton, & Starrels, 1997), and to provide personal care and assistance with activities of daily living (Kramer & Kipnis, 1995). A distinction can be drawn between gender differences in the propensity to provide care as opposed to differences in the likelihood of withdrawing from paid work, once in a caregiving role. Many studies have addressed the former, highlighting how household decisions about who provides care are subject to gendered normative societal expectations (Finch, 1989). Far fewer studies have examined gender differences in the relationship between caregiving and extended working. Zimmerman et al. (2000) showed women were more likely than men to adjust their working hours in response to caregiving demands. Others have shown caregiving commitments to negatively influence work participation only among female caregivers and not male (Carmichael & Charles, 2003; Dentinger & Clarkberg, 2002).

Our final research question tests whether these relationships are influenced by the caregiver’s hours of employment. Past research has shown caregiving outcomes to vary between full-time and part-time workers (Boaz & Muller, 1992). Caregivers working part-time might be less likely to exit work because, compared to full-time workers, they can more easily fit caregiving tasks around their working hours. On the other hand, part-time work is often associated with lower wages and lower levels of labor market attachment. Part-time workers might be more likely to exit work because the opportunity costs of caregiving are lower.

Our analyses draw upon longitudinal data for older workers (aged 50–75 years) in the United Kingdom. Many studies have examined caregiving and employment among younger cohorts, but evidence for older workers has been mixed. We contribute to this literature by considering both the hours and type of care provided. We adjust for key factors that have previously been shown to predict labor market withdrawal including age, poor self-rated health, and limiting long-term illness (van Rijn et al., 2014). We further adjust for partner’s employment status, which has been associated with early retirement (Litwin & Tur-Sinai, 2015) and may influence decisions about whether to enter into a caregiving role.

## Methods

### Data

Data were drawn from the first five waves of Understanding Society, a representative sample of the adult population (aged older than 16 years) in the United Kingdom. The sample design is described in detail elsewhere (Lynn, 2009). From the original sample, we selected 26,622 respondents who were aged 50–75 years at baseline (the wave they entered the study). From these, we excluded proxy respondents (*n* = 1,390) and those who were never working (>0 hours/week) during follow-up (2009–2015; *n* = 14,479). We further restricted the sample to individuals who responded in at least two consecutive waves, where the earlier of these was in paid employment (>0 hours/week; *n* = 2,065). The final analytical sample consisted of 8,688 individuals aged 50–75 years.

### Measures

#### Informal caregiving

Four measures of caregiving were considered: “caring intensity,” “caring location,” “relationship to care recipient,” and “change in caring status.” (a) “Caring intensity” was based on three items from the individual interviews. Respondents were first asked “Is there anyone living with you who is sick, disabled or elderly whom you look after or give special help to?” A second question asked “Do you provide regular service or help for any sick, disabled or elderly person not living with you?” If respondents answered “Yes” to either question they were then asked to estimate the number of hours spent providing care each week, both inside and outside the household. Responses were given on a 7-point scale from 0–4 to more than 100 hours/week. Due to small cell counts at the upper extremes of this scale, and to simplify interpretation, we have reduced this to three ordinal categories (0 = no care provided; 1 = 1–9 hours; 2 = more than 10 hours). The choice of 10 hours/week as the threshold for “high-intensity” caregiving was based on past research (King & Pickard, 2013; Ruhm, 1996). However, other studies have used a range of values between 10 and 20 hours/week (Carmichael & Charles, 2003; Jacobs, Laporte, van Houtven, & Coyte, 2014). We therefore tested whether our results were sensitive to the cut-point used, as detailed as follows. (b) “Caring location” was derived from the items described previously, giving a nominal variable with four categories (1 = no care provided; 2 = provides care within household; 3 = provides care outside household; 4 = provides care within and outside household). (c) “Relationship to care recipient” was derived separately for coresident and extraresident caregiving. For coresident caregiving, respondents were asked to indicate which members of the household they provided care for. We combined this with information on relationship status to derive the caring relationship. For extraresident caring, respondents were asked “Who is it that you look after or help?” The possible responses were: “Parent or parent-in-law,” “Grandparent,” “Aunt/Uncle,” “Other relatives,” “Friend or neighbor,” “Client of voluntary organization,” or “Other.” Where a respondent cared for more than one person, we took their first response only (this applied to <2% of the analytical sample). We combined information on coresident and extraresident caregiving to give four categories (0 = no care provided; 1 = partner/spouse; 2 = parent/grandparent; 3 = other). More detailed categorizations were not possible due to low cell counts. (d) “Change in caring status” was derived based on caregiving across two consecutive waves (T_n_ and T_n+1_):

1. Noncaregiver (not providing care at T_n_ and T_n+1_; 0 hours/week)
2. Past caregiver (providing care at T_n_, but not T_n+1_; from >0 to 0 hours/week)
3. Continuing caregiver (providing care both T_n_ and T_n+1_)
4. New caregiver (not providing care at T_n_, but starts providing care by T_n+1_)

For “continuing” and “new” caregivers, we further split these categories based on the hours of care provided (1–9 vs. more than 10 hours/week).

#### Work exit

Exit from paid employment was defined as a reduction in working hours across two consecutive waves, from >0 hours/week (at T_n_) to 0 hours/week (at T_n+1_, 1 year later). Weekly working hours were based on hours worked in the respondent’s main and second job and included overtime.

#### Covariates

Models were adjusted for continuous age, age-squared, self-rated general health (“Excellent,” “Very good,” “Good,” “Fair,” or “Poor”), long-standing illness, or disability (>12 months; 0 = No; 1 = Yes), occupation derived from the Standard Occupational Classification 2000 (1 = Professional; 2 = Intermediate; 3 = Nonmanual; 4 = Manual/other), and partner’s employment status (1 = No partner; 2 = Partner not working; 3 = Partner is working).

### Analytical Approach

We used a discrete-time survival model to estimate associations between caregiving and work exit (Steele, Goldstein, & Browne, 2004). This modeled the probability that an individual stops working between the current interview (T_n_) and their next interview (T_n+1_, the following year), conditional on them being in work at T_n_ (>0 hours/week). For this analysis, we restructured the data as follows. First, we defined baseline for each individual as the first wave they are in paid work (>0 hours/week). Second, for each wave that an individual was in paid work (including baseline) we defined a binary outcome indicating whether they had stopped working (0 hours/week; y = 1) or continued working (>0 hours/week; y = 0) by the subsequent interview, 1 year later (T_n+1_). Repeated observations for each individual were thus represented as a sequence of 0s, ending with a value of 1 (where an exit from work was observed) or 0 (where the individual was right-censored). Having restructured the data, we fitted a random intercept logistic regression model in Stata 14.1 (StataCorp, 2015) with repeated observations nested within individuals. This modeled the log of the hazard ratio as a linear function of the covariates. This approach allowed for multiple episodes, where a respondent could return to work having previously exited (e.g., two sequences of {0, 1} and {0, 1}; this applied to just 338 respondents). All models were adjusted for sample design (stratification and primary sampling unit) and weighted using weights provided by Understanding Society that adjusted for unequal selection probabilities, differential nonresponse, and potential sampling error.

We estimated all models for women and men separately. We further stratified by weekly working hours, estimating results for part-time (1–29 hours) and full-time (more than 30 hours) employees separately.

### Sensitivity Tests

We considered reductions in working hours as an alternative to work exit. This was tested using discrete-time survival models, described previously, but expanded to allow for competing risks. Between each interview, an individual could either continue working (*y* = 0), reduce their hours (from more than 30 to 1–29 hours/week; *y* = 1), or exit work altogether (0 hours/week; *y* = 2). The response variable for each respondent thus represented a sequence of 0s followed by 1 or 2. We modeled this multinomial outcome using a multilevel multivariate model estimated in Stata 14.1.

We further tested whether the relationship between caregiving and paid work was modified by age, poor self-rated health, or hourly wage. Hourly wage was derived based on self-reported weekly earnings divided by the number of hours worked (in all jobs, including overtime). We also tested sensitivity to the 10-hour cut-point for caregiving intensity, by respecifying the analysis with a range of alternative cut-points for “high-intensity” caregiving (10, 15, and 20 hours/week).

## Results

The analytical sample consisted of 20,959 responses from 8,473 individuals. Individuals were omitted due to missing information on caregiving status (*n* = 199), long-term limiting illness (*n* = 1), self-rated health (*n* = 1), and occupation (*n* = 14). Compared to respondents not working at baseline, the analytical sample is younger (average age = 55.7 vs. 62.7; *p* < .0001) and less likely to report poor self-rated health (2.7% vs. 13.7%; *p* < .0001) or long-standing limiting illness (36.6% vs. 54.0%; *p* < .0001).

Table 1 presents characteristics of the analytical sample at baseline (the wave the participant was first included in the analysis). Most respondents were working full time (68.9%) and provided no informal care (76.5%). Women were more likely to provide low- (1–9 hours) and high-intensity (more than 10 hours) caregiving, compared to men, and were less likely to provide no care. Women working full time were almost twice as likely to provide high-intensity care compared to men (8.9% vs. 4.7%), but this difference was not found between women and men working part time.

**Table 1.** Characteristics of the Analytical Sample at Baseline^a^

	Total sample	Part-time (1–29 hours)		Full-time (30+ hours)		Δ groups
		Women	Men	Women	Men	
	(*n* = 8,473)	(*n* = 1,873)	(*n* = 550)	(*n* = 2,714)	(*n* = 3,336)	
Age (years)						
M	55.65	56.80	60.53	54.20	55.35	F(3) = 166.0***
SD	5.33	6.03	6.44	4.38	4.76	
Weekly working hours						
M	35.69	18.55	17.68	40.28	44.10	F(3) = 4928.8***
SD	13.88	6.60	6.55	8.80	9.31	
	N (%)	N (%)	N (%)	N (%)	N (%)	
Self-rated health						
Excellent	1,456 (17.2)	317 (16.8)	86 (16.0)	528 (19.7)	525 (15.7)	
Very good	2,956 (34.9)	649 (35.1)	189 (34.0)	934 (34.9)	1,184 (34.9)	
Good	2,700 (31.8)	580 (31.0)	169 (30.6)	836 (29.6)	1,115 (34.0)	
Fair	1,140 (13.5)	269 (14.1)	78 (13.3)	347 (13.1)	446 (13.6)	
Poor	221 (2.7)	58 (3.1)	28 (6.1)	69 (1.9)	66 (2.7)	Χ2(12) = 59.6***
Long-standing illness	3,036 (36.6)	706 (37.7)	219 (41.4)	962 (36.4)	1,149 (35.4)	Χ2(3) = 2.3
Partner’s employment status						
No partner	3,271 (35.3)	691 (34.9)	187 (31.7)	1,227 (42.2)	1,164 (31.0)	
Partner not working	1,203 (14.2)	271 (14.8)	116 (20.5)	274 (9.5)	542 (16.3)	
Partner is working	3,999 (50.5)	910 (50.4)	247 (47.8)	1,213 (48.3)	1,629 (52.7)	Χ2(6) = 129.6***
Occupation						
Professional	1,873 (22.8)	141 (7.5)	103 (19.0)	598 (22.4)	1,031 (31.7)	
Intermediate	2,079 (24.4)	421 (22.8)	104 (19.4)	917 (33.7)	637 (19.1)	
Nonmanual	2,019 (23.6)	845 (45.9)	121 (22.6)	743 (27.8)	310 (9.2)	
Manual/other	2,502 (29.2)	466 (23.8)	222 (39.0)	456 (16.1)	1,358 (40.0)	Χ2(9) = 1477.1***
Weekly hours of caregiving						
0	6,483 (76.5)	1,376 (73.5)	431 (77.8)	1,969 (72.3)	2,707 (80.8)	
0–9	1,331 (16.1)	289 (16.4)	71 (13.1)	498 (18.9)	473 (14.4)	
>10	659 (7.5)	208 (10.1)	48 (9.1)	247 (8.9)	156 (4.7)	Χ2(6) = 99.3***
Location of caregiving						
No care provided	6,483 (76.5)	1,376 (73.5)	431 (77.8)	1,969 (72.3)	2,707 (80.8)	
Within household	409 (4.6)	104 (5.3)	37 (6.8)	125 (4.3)	143 (4.1)	
Outside household	1,474 (17.6)	365 (19.7)	77 (14.1)	580 (21.8)	452 (14.0)	
Both	107 (1.4)	28 (1.5)	5 (1.3)	40 (1.6)	34 (1.1)	Χ2(9) = 90.9***
Relationship to care recipient						
No caring	6,483 (76.5)	1,376 (73.5)	431 (77.8)	1,969 (72.3)	2,707 (80.8)	
Partner/spouse	207 (2.3)	59 (2.9)	19 (3.8)	60 (2.0)	69 (1.9)	
Parent/grandparent	1,217 (14.7)	287 (15.8)	56 (10.7)	483 (18.2)	391 (12.2)	
Other	566 (6.6)	151 (7.7)	44 (7.7)	202 (7.6)	169 (5.1)	Χ2(9) = 94.2***
Change in caring status						
Noncarer	5,820 (68.6)	1,201 (64.0)	384 (69.8)	1,748 (63.8)	2,487 (74.3)	
Past carer	633 (7.5)	140 (7.9)	35 (6.4)	223 (8.1)	235 (7.0)	
Continuing (<10 hours)	807 (9.8)	175 (9.5)	34 (6.7)	328 (12.8)	272 (8.4)	
Continuing (10+ hours)	493 (5.6)	160 (8.0)	32 (6.6)	189 (6.6)	112 (3.6)	
New carer (<10 hours)	485 (5.8)	116 (6.2)	30 (4.7)	165 (6.7)	173 (5.2)	
New carer (10+ hours)	235 (2.7)	81 (4.4)	35 (5.9)	61 (2.1)	57 (1.6)	Χ2(15) = 134.6***

*Note:* ***p < .001.

^a^Baseline is defined as the wave when the participant was first included in the analysis.

Tables 2 and 3 present the odds ratios for exit from work during follow-up for women and men, respectively. These models were stratified by working hours and adjusted for age, self-rated health, long-standing illness or disability, partner’s employment status and occupation. For both women and men, we found no evidence of an association between the hours of care provided and subsequent exit from work, irrespective of working hours. For full-time workers, the location of caring and relationship to care recipient were associated with exit from work. Women and men providing care within the household were 1.93 (95% confidence interval [CI]: 1.23–3.01) and 1.54 (95% CI: 1.07–2.21) times more likely to exit paid employment, compared to noncaregivers. Women caring for a partner or spouse were more likely to stop working, compared to noncaregivers (odds ratio [OR] = 1.97; 95% CI: 1.10–3.51) but this association was not observed among men.

**Table 2.** Odds Ratios for Women for Exit From Work (0 hour/week) by Subsequent Wave, Based on Caregiving This Wave

	PT (Model 1)	FT (Model 2)	PT (Model 3)	FT (Model 4)	PT (Model 5)	FT (Model 6)
	OR [95% CI]	OR [95% CI]	OR [95% CI]	OR [95% CI]	OR [95% CI]	OR [95% CI]
Weekly hours of care provided						
0	1.00 [1.00, 1.00]	1.00 [1.00, 1.00]				
1–9	0.92 [0.70, 1.21]	0.79 [0.58, 1.08]				
10+	0.87 [0.61, 1.23]	1.03 [0.72, 1.49]				
Location of caregiving						
No care provided			1.00 [1.00, 1.00]	1.00 [1.00, 1.00]		
Within household			0.92 [0.58, 1.46]	1.93 [1.23, 3.01]**		
Outside household			0.93 [0.72, 1.20]	0.70 [0.52, 0.93]*		
Both			0.48 [0.19, 1.22]	0.73 [0.26, 2.02]		
Relationship to care recipient						
No caring					1.00 [1.00, 1.00]	1.00 [1.00, 1.00]
Partner or spouse					1.03 [0.60, 1.76]	1.97 [1.10, 3.51]*
Parent or grandparent					0.89 [0.66, 1.19]	0.82 [0.60, 1.12]
Other					0.88 [0.60, 1.28]	0.70 [0.44, 1.10]
Individuals	1,873	2,714	1,873	2,714	1,873	2,714

*Note:* Adjusted for age, self-rated health, long-term limiting illness, occupation, and partner’s employment status. Each measure of caregiving is tested separately, stratified by working hours. CI = confidence interval; FT = full-time (more than 30 hours/week); OR = odds ratio; PT = part-time (1–29 hours/week).

*p < .05, **p < .01.

**Table 3.** Odds Ratios for Men for Exit From Work (0 hour/week) by Subsequent Wave, Based on Caregiving This Wave

	PT (Model 1)	FT (Model 2)	PT (Model 3)	FT (Model 4)	PT (Model 5)	FT (Model 6)
	OR [95% C.I.]	OR [95% C.I.]	OR [95% C.I.]	OR [95% C.I.]	OR [95% C.I.]	OR [95% C.I.]
Weekly hours of care provided						
0	1.00 [1.00, 1.00]	1.00 [1.00, 1.00]				
1–9	0.94 [0.58, 1.51]	1.01 [0.79, 1.29]				
10+	0.92 [0.48, 1.80]	1.35 [0.95, 1.91]				
Location of caregiving						
No care provided			1.00 [1.00, 1.00]	1.00 [1.00, 1.00]		
Within household			1.10 [0.50, 2.42]	1.54 [1.07, 2.21]*		
Outside household			0.91 [0.57, 1.47]	1.00 [0.79, 1.27]		
Both			0.31 [0.03, 3.76]	0.83 [0.31, 2.20]		
Relationship to care recipient						
No caring					1.00 [1.00, 1.00]	1.00 [1.00, 1.00]
Partner or spouse					0.71 [0.27, 1.88]	1.41 [0.86, 2.29]
Parent or grandparent					0.98 [0.55, 1.76]	1.07 [0.83, 1.39]
Other					0.99 [0.51, 1.93]	1.07 [0.76, 1.50]
Individuals	550	3,336	550	3,336	550	3,336

*Note:* Adjusted for age, self-rated health, long-term limiting illness, occupation, and partner’s employment status. Each measure of caregiving is tested separately, stratified by working hours. CI = confidence interval; FT = full-time (more than 30 hours/week); OR = odds ratio; PT = part-time (1–29 hours/week).

*p < .05.

Table 4 presents results for changes in caregiving status. Women who started providing high-intensity care (from 0 hours/week at T_n_ to more than 10 hours at T_n+1_) were between 2.6 and 4.5 times more likely to have stopped working by their next interview, compared to noncaregivers (OR for part-time = 2.64; 95% CI: 1.46–4.79; OR for full-time = 4.46; 95% CI: 2.53–7.88). No statistically significant associations were observed for men, nor for women maintaining their hours of caregiving or starting a caregiving role at lower intensity (1–9 hours/week).

**Table 4.** Odds Ratios for Work Exit (0 hour/week) by Next Wave (T_n+1_) Based on Change in Caregiving Status (Between T_n_ and T_n+1_)

		Women		Men	
		PT (Model 1)	FT (Model 2)	PT (Model 3)	FT (Model 4)
		OR [95% C.I.]	OR [95% C.I.]	OR [95% C.I.]	OR [95% C.I.]
Change in caring status					
Noncarer		1.00 [1.00, 1.00]	1.00 [1.00, 1.00]	1.00 [1.00, 1.00]	1.00 [1.00, 1.00]
Past carer		0.81 [0.56, 1.17]	1.10 [0.75, 1.63]	1.00 [0.53, 1.87]	1.04 [0.77, 1.42]
Continuing (<10 hours)		1.03 [0.74, 1.43]	0.84 [0.57, 1.25]	0.71 [0.38, 1.35]	1.08 [0.81, 1.45]
Continuing (10+ hours)		0.95 [0.64, 1.41]	1.02 [0.65, 1.60]	1.25 [0.58, 2.72]	1.38 [0.94, 2.03]
New carer (<10 hours)		0.92 [0.61, 1.38]	1.35 [0.85, 2.14]	1.08 [0.55, 2.13]	0.97 [0.69, 1.38]
New carer (10+ hours)		2.64 [1.46, 4.79]**	4.46 [2.53, 7.88]***	1.24 [0.41, 3.75]	1.60 [0.90, 2.83]
Individuals		1,873	2,714	550	3,336

*Note:* Adjusted for age, self-rated health, long-term limiting illness, occupation, and partner’s employment status. Coefficients estimated separately for women and men, stratified by working hours. Each measure of caregiving is tested separately, stratified by working hours. CI = confidence interval; FT = full-time (more than 30 hours/week); OR = odds ratio; PT = part-time (1–29 hours/week).

**p < .01, ***p < .001.

### Sensitivity Analyses

Supplementary Table 1 (available online) considers reductions in working hours as an alternative to work exit. These models were estimated for full-time workers only, stratified by sex and adjusted for the covariates described previously. The results for “stopping work” (from more than 30 hours/week at T_n_ to 0 hours at T_n+1_) were consistent with the simpler models described previously. Women providing low-intensity care (OR = 1.46; 95% CI: 1.06–2.00), extraresidential care (OR = 1.50; 95% CI: 1.10–2.04), or care for a parent/grandparent (OR = 1.54; 95% CI: 1.11–2.15) were more likely to reduce their working hours, compared to women providing no care. Women and men who maintained a low-intensity caregiving role (1–9 hours) were also more likely to reduce their working hours (OR for women = 1.60; 95% CI: 1.08–2.36; OR for men = 1.75; 95% CI: 1.06–2.89).

We found no evidence of effect modification by age, poor self-rated health, or hourly wage. The cut-point used for “high-intensity” caregiving did not influence the direction or statistical significance of the results.

## Discussion

Among a nationally representative sample of older workers, we identified groups of caregivers at risk of exiting paid employment or reducing working hours. Women who entered a high-intensity caregiving role were more likely to stop working, compared to women providing no care. Full-time employees who provided coresident care (women and men) or care for a partner/spouse (women only) were also more likely to stop working, compared to those not providing care, but this association was not observed among part-time employees. Among women, low-intensity care, extraresidential care, and care for a parent/grandparent were associated with reduced working hours.

Our analysis highlights the onset of caregiving as a key period in explaining labor market transitions among women. Older workers who continue an existing caregiving role have already overcome the challenges of combining work and care (for example, by adjusting work schedules, reducing work demands, or seeking support from family or friends). Those entering a caregiving role, by contrast, are at greater risk of leaving work because they have yet to successfully balance these competing demands. This is consistent with past evidence. King and Pickard (2013) similarly found older women in England entering a high-intensity caregiving role (more than 10 hours/week) to be at greater risk of stopping work, as did Carr and Kail (2013) for older workers in the United States. Our findings can also be compared to Carmichael et al. (2010), who found similar results based on the British Household Panel Survey, the predecessor to Understanding Society. Their study found the onset of high-intensity caregiving to be associated with exit from paid work, but using a different cut-point for high-intensity care (20 hours/week) and for workers aged 19–64 years (compared to ages 50–75 in our analysis).

We found no evidence for an association between hours of care provided and exit from paid work. This is consistent with some studies (Leigh, 2010) but contradicts others (Harper, 2004). While mixed results may reflect contextual differences between study populations, the inconsistent measurement of caregiving is another likely source of discrepancy. Most large surveys, including Understanding Society, collect only basic information about the amount and type of care provided, yet past studies have shown the characteristics of caregiving to be important for subsequent employment outcomes (van Houtven et al., 2013). Like us, Leigh (2010) found no association between caregiving and work participation based on the “hours of care provided.” By contrast, van Houtven et al. (2013) and Harper (2004) found significant associations, but only for measures of personal care (e.g., dressing, bathing); other forms of caregiving had no effect. Inconsistent results for the caring–working relationship may therefore reflect inconsistent measurement of caregiving status and caregiving demands.

Our results suggest that the impact of caregiving on paid work at older ages depends upon the type of the care provided. Among full-time workers, coresident caregiving was associated with increased risk of work exit, consistent with past studies (Corti et al., 1994; Heitmueller & Michaud, 2006). Among women, care for a partner/spouse was associated with exit from work, but no associations were observed for other caregiving relationships. Partner/spousal care is likely to coincide with coresident caregiving. Since we found no association between hours of caregiving and work exit, this suggests that the nature of coresident caregiving (such as personal care or other time-sensitive tasks) or the emotional costs of caring for a partner/spouse (Tuithof, ten Have, van Dorsselaer, & de Graaf, 2015) may result in stronger employment impacts for these types of caregiving, compared to other roles.

We found differences between women and men, in line with past studies (Carmichael & Charles, 2003; Dentinger & Clarkberg, 2002; Zimmerman et al., 2000). Care for a partner/spouse was associated with work exit among women only, as was the onset of a high-intensity caregiving role. Women were also more likely to reduce their working hours in response to caregiving demands, compared to men. Several factors may explain these differences. Women’s propensity to provide care is shaped by “the social construction of gender, traditional family roles, and societal constructs including economic arrangements” (Laditka & Laditka, 2001, p. 432). Women curtail employment because they expect, and are expected to, fulfill sex role-typical employment behaviors. Varying statutory pension ages (of 60 and 65 for women and men in our sample, respectively) are another potential explanation (King & Pickard, 2013). Women leaving the labor market early, compared to men of similar age, will be closer to the age at which they can draw their pension and would experience less negative impact on future pension entitlements. Another possibility is that women and men respond differently to caregiving demands. Dentinger and Clarkberg (2002) found that whereas women tended to stay at home and provide care for an ill or disabled family member, men responded by delaying retirement, instead “shouldering the financial burden associated with disability” (p. 876).

Our results also differed by working hours. With one exception, we found no associations between caregiving and work withdrawal among part-time employees, consistent with past studies (Boaz & Muller, 1992). This suggests that part-time workers can more easily fit caregiving tasks around work commitments, and counters the earlier notion that lower opportunity costs among part-time workers lead to stronger associations between caregiving and work withdrawal.

### Strengths and Limitations

Our analysis drew upon a nationally representative, longitudinal sample of older workers. Few studies have examined caregiving and paid work among older workers in the United Kingdom, and none to our knowledge using the latest waves of Understanding Society. We were able to adjust for several potential confounders, and repeated measures of caring and working hours made it possible to test longitudinal relationships between caregiving and work exit. We were able to stratify the analysis by sex and working hours, and assessed the type of caregiving role in addition to the hours of care provided.

Regarding limitations, we relied on a basic measure of caregiving intensity and could not consider more detailed caregiving characteristics (e.g., personal care vs. help with chores) or the type of health condition being cared for. Although our findings were not sensitive to the chosen threshold for high-intensity caregiving, hours of care represent just one aspect of the overall caregiving burden. Other indicators—such as being the primary caregiver (Nguyen & Connelly, 2014), providing personal care (Boaz & Muller, 1992), or caring for individuals with dementia (Ory, Hoffman, Yee, Tennstedt, & Schulz, 1999)—are important in determining employment outcomes. Besides working hours, we were unable to include measures of the psychosocial work environment (Kubicek et al., 2010). Our analysis did not consider retirement status, and instead, defined work exit based on a reduction in working hours. We considered this preferable to self-reported retirement status, which incorporates many factors besides hours of paid work (e.g., end of main career job or receipt of statutory pension). Finally, individuals in the analytical sample were younger and healthier than respondents not working at baseline, and were less likely to work in manual occupations. Our analysis, therefore, is representative of older workers, but not those who have left the labor market before age 50.

An aging workforce is expected to remain in work while simultaneously expanding the supply of informal care. Achieving these goals in parallel will require interventions that facilitate the combination of paid and unpaid roles. Our results contribute to a growing evidence-base highlighting incompatibilities between caregiving and later-life employment. Improving recognition and support for older workers around the onset of caregiving may reduce the likelihood of subsequent withdrawal from work. Many governments are seeking to extend working life by raising statutory retirement ages, with considerable effect. In the United Kingdom, employment rates among those aged 50–64 years rose from 64.7% in 2006 to 70.7% in 2016 (Office for National Statistics, 2016). However, there is no equivalent policy lever when it comes to social care. The employment impacts of caregiving will depend upon individual, family- and work-related circumstances. Policy should reflect individual needs, with particular attention given to women and individuals combining caregiving with full-time employment. Flexible working arrangements or increased job control may make it easier to balance competing demands, but further research is required to understand the role of workplace characteristics in the caring–working relationship.

## Supplementary Material

Please visit the article online at http://psychsocgerontology.oxfordjournals.org/ to view supplementary material.

## Funding

This work was supported by the Economic and Social Research Council and the Medical Research Council as part of the Lifelong Health and Well-Being initiative (grant number ES/L002892/1); and the UK Medical Research Council (grant number MRC_MC_UU_12019/5 to M.S.).

## Conflict of interest

The authors declare that they have no conflict of interest.

## Supplementary Material

Supplementary_Table_1
