# Supplementary material for: The Association Between Informal Caregiving and Exit From Employment Among Older Workers: Prospective Findings From the UK Household Longitudinal Study
Source: J Gerontol B Psychol Sci Soc Sci. 2016 Dec 6;73(7):1253–62. doi: 10.1093/geronb/gbw156 (PMC6146784; doi:10.1093/geronb/gbw156)
Supplement: Supplementary_Table_1 [file gbw156_suppl_supplementary_table_1.docx]

| **Supplementary Table 1: Odds ratios for competing risks survival analysis (reduced working hours vs. exit from work)**  **for full-time workers only (n = 6050)** | | | | | | | | | |
| --- | --- | --- | --- | --- | --- | --- | --- | --- | --- |
|  |  | Women (Model 1) | | Men (Model 2) | | Women (Model 3) | | Men (Model 4) | |
|  |  | Reduce | Stop | Reduce | Stop | Reduce | Stop | Reduce | Stop |
|  |  | OR [95% CI] | OR [95% C.I.] | OR [95% C.I.] | OR [95% C.I.] | OR [95% C.I.] | OR [95% C.I.] | OR [95% C.I.] | OR [95% C.I.] |
| *Weekly hours of care provided* | | | | | | | | | |
|  | 0 | 1.00 | 1.00 | 1.00 | 1.00 |  |  |  |  |
|  |  | [1.00, 1.00] | [1.00, 1.00] | [1.00, 1.00] | [1.00, 1.00] |  |  |  |  |
|  | 1-9 | 1.46* | 0.80 | 1.39 | 0.98 |  |  |  |  |
|  |  | [1.06, 2.00] | [0.61, 1.05] | [0.93, 2.06] | [0.81, 1.20] |  |  |  |  |
|  | 10+ | 1.05 | 1.05 | 1.32 | 1.27 |  |  |  |  |
|  |  | [0.67, 1.64] | [0.75, 1.46] | [0.71, 2.49] | [0.97, 1.67] |  |  |  |  |
| *Location of caregiving* | | | | | | | | | |
|  | No care provided | | | | | 1.00 | 1.00 | 1.00 | 1.00 |
|  |  | | | | | [1.00, 1.00] | [1.00, 1.00] | [1.00, 1.00] | [1.00, 1.00] |
|  | Within household | | | | | 0.70 | 1.82** | 1.10 | 1.51* |
|  |  | | | | | [0.35, 1.40] | [1.23, 2.69] | [0.53, 2.28] | [1.08, 2.10] |
|  | Outside household | | | | | 1.50** | 0.71* | 1.34 | 1.00 |
|  |  | | | | | [1.10, 2.04] | [0.55, 0.93] | [0.91, 1.99] | [0.80, 1.26] |
|  | Both | | | | | 0.83 | 0.71 | 3.70* | 0.84 |
|  |  |  |  |  |  | [0.29, 2.38] | [0.30, 1.70] | [1.20, 11.36] | [0.33, 2.14] |
|  |  | Women (Model 5) | | Men (Model 6) | | Women (Model 7) | | Men (Model 8) | |
|  |  | Reduce | Stop | Reduce | Stop | Reduce | Stop | Reduce | Stop |
| *Relationship to care recipient* | | | | | | | | | |
|  | No caring | 1.00 | 1.00 | 1.00 | 1.00 |  |  |  |  |
|  |  | [1.00, 1.00] | [1.00, 1.00] | [1.00, 1.00] | [1.00, 1.00] |  |  |  |  |
|  | Partner/spouse | 0.85 | 1.84* | 1.29 | 1.37 |  |  |  |  |
|  |  | [0.34, 2.12] | [1.09, 3.08] | [0.52, 3.23] | [0.87, 2.17] |  |  |  |  |
|  | Parent/grand-parent | 1.54** | 0.83 | 1.43 | 1.07 |  |  |  |  |
|  |  | [1.11, 2.15] | [0.63, 1.09] | [0.92, 2.22] | [0.83, 1.38] |  |  |  |  |
|  | Other | 0.99 | 0.73 | 1.31 | 1.07 |  |  |  |  |
|  |  | [0.61, 1.63] | [0.49, 1.10] | [0.69, 2.46] | [0.77, 1.48] |  |  |  |  |
| *Change in caring status* | | | | | | | | | |
|  | Non-carer | | | |  | 1.00 | 1.00 | 1.00 | 1.00 |
|  |  | | | |  | [1.00, 1.00] | [1.00, 1.00] | [1.00, 1.00] | [1.00, 1.00] |
|  | Past carer | | | |  | 1.27 | 1.06 | 1.16 | 1.04 |
|  |  | | | |  | [0.83, 1.96] | [0.74, 1.53] | [0.66, 2.02] | [0.77, 1.41] |
|  | Continuing (<10h) | | | |  | 1.60* | 0.84 | 1.75* | 1.08 |
|  |  | | | |  | [1.08, 2.36] | [0.60, 1.17] | [1.06, 2.89] | [0.81, 1.43] |
|  | Continuing (10+h) | | | |  | 1.01 | 1.03 | 0.95 | 1.36 |
|  |  | | | |  | [0.60, 1.70] | [0.70, 1.51] | [0.41, 2.19] | [0.94, 1.97] |
|  | New carer (<10h) | | | |  | 0.93 | 1.29 | 1.01 | 0.97 |
|  |  | | | |  | [0.54, 1.60] | [0.86, 1.94] | [0.54, 1.86] | [0.69, 1.36] |
|  | New carer (10+h) | | | |  | 1.34 | 3.74*** | 0.42 | 1.58 |
|  |  |  |  |  |  | [0.54, 3.30] | [2.29, 6.11] | [0.07, 2.45] | [0.90, 2.77] |
|  |  | 2714 | | 3336 | | 2714 | | 3336 | |
|  | *Notes.* Adjusted for age, self-rated health, long-term limiting illness, occupation, and partner's employment status. Coefficients for women, men, part-time (PT) and full-time (FT) workers estimated separately. CI = confidence interval. * p<0.05, ** p<0.01, *** p<0.001 | | | | | | | | |
